# Different SARS-CoV-2 variants inhibited by RRM designed peptide

**DOI:** 10.1371/journal.pone.0327582

**Published:** 2025-07-22

**Authors:** Uros Krapez, Urska Kuhar, Petra Šenica, Brigita Slavec, Drasko Cosic, Ivan Loncarevic, Sandra Janezic, Irena Cosic

**Affiliations:** 1 Institute for Poultry, Birds, Small Mammals, and Reptiles, Veterinary Faculty, University of Ljubljana, Ljubljana, Slovenia; 2 Institute of Microbiology and Parasitology, Veterinary Faculty, University of Ljubljana, Ljubljana, Slovenia; 3 AMALNA Consulting, Black Rock, Melbourne, VIC, Australia; 4 QuantBioRes - QBR A/S, Copenhagen, Denmark; 5 National Laboratory for Health, Environment and Food, Maribor, Slovenia; University of Coimbra: Universidade de Coimbra, PORTUGAL

## Abstract

The SARS-CoV-2 virus has mutated over time, resulting in variations of circulating viral variants, which may impact the virus’s properties, such as transmission or the severity of symptoms. Thus, there is a need for comprehensive approach less dependent of viral variants. For that purpose, we have employed the innovative and unique Resonant Recognition Model (RRM) to identify the common characteristics of all variants of SARS-CoV-2 virus and based on these characteristics, we have explored the possibility to develop approach against SARS-CoV-2 infection, less dependent on viral variants. This paper is a continuation of our previous research, where we have used the RRM model to design *de novo* peptide CovA, capable to prevent SARS-CoV-2 interaction with ACE2 receptor on host cells. Using Inhibitor Screening Assay Kits and viral replication in SARS-CoV-2/Vero E6 cells model, it has been previously shown that CovA can prevent viral interaction with receptor and viral replication in host cells. This test has been done on Wuhan variant only. Here, we have tested this RRM designed peptide CovA for efficiency with some other different SARS-CoV-2 variants. It has been shown here, that CovA act on all tested viral variants, but with different efficiency. Apart from representing the basis of new COVID-19 drugs discovery, this research once again presents the ability of RRM model to design *de novo* bioactive peptides with desired biological function.

## Introduction

The SARS-CoV-2 virus, which causes COVID-19 disease, has mutated over time, resulting in genetic variation within the population of circulating viral variants. This genetic variation may impact the virus’s properties such as transmission (for example, it may spread more easily) or the severity of symptoms on infected individuals (for example, it may cause more severe disease) [1]. Mutations in the SARS-CoV-2 virus have introduced a challenge in preventing and treating COVID-19 disease. Different mutations are producing new viral variants, which could be of particular importance due to their potential for increased transmissibility [2], increased virulence, or reduced effectiveness of vaccines against them [3]. There is evidence from laboratory studies that some immune responses driven by current vaccines could be less effective against some of these variants [1], which may contribute to the continuation of the COVID-19 infections.

The Alpha, Delta and Omicron variants marked different phases in the development of the COVID-19 pandemic. Each variant brought new challenges in terms of viral spread, clinical outcomes and public health response. The Alpha variant has a significant mutation N501Y in the spike (S) protein, which could be responsible for an approximately 50% increase in transmissibility compared to the original Wuhan variant. The mutation in the S protein of the Alpha variant had little effect on the efficacy of vaccines based on an original strain. The Delta variant, which was discovered later in the same year 2020 as the Alpha variant, has two key mutations in the S protein (L452R and P681R respectively), which resulted in higher transmissibility and lower vaccine efficacy compared to the Alpha variant [4]. The main public health impact caused by the emergence and global spread of the aforementioned SARS-CoV-2 variants was a higher risk of hospitalisation and increased mortality [4]. On the other hand, the Omicron variant, which was discovered in 2021, has over 30 mutations in the S protein compared to the Wuhan variant, resulting in lower vaccine efficacy and lower neutralisation of antibodies in convalescents [5]. In addition, the Omicron variant is much more transmissible than the previously discovered SARS-CoV-2 variants, although the clinical symptoms are usually milder compared to those of the Alpha or Delta variants [6].

Due to the continuous evolution of SARS-CoV-2, there seems to be a need for a more comprehensive approach that is less reliant on specific viral variants. For that purpose, we have employed the Resonant Recognition Model (RRM) to identify the common characteristics of all variants of SARS-CoV-2 virus and based on these characteristics, we have explored the possibility to develop approach against SARS-CoV-2 infection less dependent on virus mutations.

The RRM is innovative approach using knowledge from quantum physics, biochemistry and signal processing to analyse protein biological functions/interactions. As such, it is capable to predict bioactive mutations in proteins and DNA/RNA, and even more to design *de novo* bioactive peptides with desired biological function [7–9]. The RRM model is extensively published and experimentally tested in our previous research [7–17]. In particular, the design of *de novo* peptides with desired biological function could be used for design of novel peptidic drugs with targeted biological function including: peptide that against resistant bacteria [10], peptide that can prevent SARS-CoV-2 virus entry into the host cells via ACE2 receptor [11–12], IL-12 analogue [13], peptide to mimic myxoma virus oncolytic function [14], HIV envelope protein analogue [15,16], FGF analogue [17].

This paper is continuation of our previous research, where we have, using the RRM approach, designed *de novo* 18-mer peptide CovA capable of preventing SARS-CoV-2 interaction with ACE2 receptor on host cells [11–12]. Within that previous research we have designed *de novo* peptide CovA, which has been shown experimentally to influence interaction between SARS-CoV-2 spike proteins (Wuhan variant) and ACE2 receptors on Vero E6 host cells, using Inhibitor Screening Assay Kits, as well as in SARS-CoV-2/Vero E6 cells model [11].

**Fig 1:**
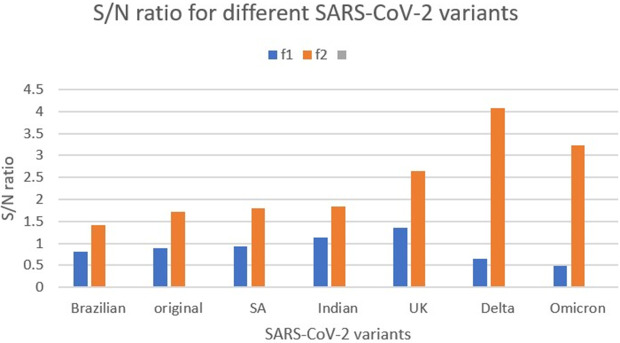
RRM parameters for different SARS-CoV-2 variants. S/N ratio at RRM characteristic frequency f1 in blue for whole spike protein and frequency f2 in orange for S1 spike fragment for different SARS-CoV-2 variants. It can be observed that more virulent variants have higher S/N ratio at RRM characteristic frequencies f2 (orange bars) and that strength of virus variant is correlated with S/N ratio at RRM characteristic frequencies f1 (blue bars) [18–20].

Although all SARS-CoV-2 variants have the same RRM characteristic parameters in common, we have analysed the strength of these parameters in different viral variants, and we have found the strong correlation between amplitudes (S/N ratio) of RRM parameters (f1 and f2) with viral strength and viral infectivity respectively [18–20], as presented in Fig 1. More detailed explanations including SARS-CoV-2 variants nomenclature and raw data regarding Fig 1 are presented within supporting information file S1 File.

As peptide CovA was designed from common RRM characteristics of different SARS-CoV-2 variants, it is expected that it will be effective with other viral variants. This *de novo* designed peptide CovA, has been previously tested on Wuhan variant only, but here we have experimentally tested its effectiveness against other different variants of SARS-CoV-2 virus, including Alpha, Delta, Wuhan and Omicron.

## Results

**Fig 2:**
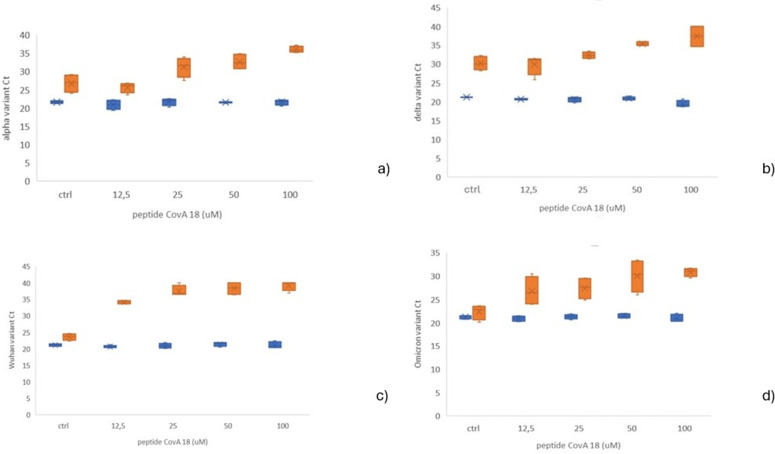
Ct values forviral RNA for different SARS-CoV-2 variants. Ct values (PCR threshold cycles) are presented for viral RNA in orange and GAPDH in blue as control for variants: a) Alpha, b) Delta c) Wuhan and d) Omicron. It can be observed that RRM designed peptide CovA prevent Alpha, Delta, Wuhan and Omicron infection and replication in the Vero E6 cell culture model, but with different efficiency.

Initially, it has been tested how peptide CovA affects amounts of viral RNA detected in Vero E6 cells after infection with Alpha, Delta, Wuhan and Omicron variants of SARS-CoV-2 virus. For that purpose, the peptide diluted in EMEM (Eagle’s Minimum Essential Medium) was added to the monolayer of washed Vero E6 cells and pre-incubated. After pre-incubation, cells were infected with SARS-CoV-2 variants: Alpha, Delta, Wuhan and Omicron. The virus-peptide mixture was removed, and peptide was added to the cells and incubated for 18h. All incubations were performed at 37°C in 5%CO2 incubator. Viral RNA was isolated and measured using real-time RT-qPCR assay targeting the E gene of SARS-CoV-2 and GAPDH as endogenous control. Results are shown as Ct values (single data points derived from real-time PCR amplification plots also known as threshold cycles) for RNA viral loads of different tested SARS-CoV-2 variants with GAPDH expression used as control. Possible outliers were identified on ∆Ct values with IQR method and excluded. The highest differences in Ct values between controls and peptide CovA treated cells suggests exponentially higher reduction in the concentration of the viral RNA. It can be observed from Fig 2 that peptide CovA is the most potent in reduction of viral infection and replication for Wuhan variant compared to other variants. However, for all variants peptide CovA is reducing for several hundred folds concentration of the viral RNA indicating that it is significantly, but with different efficiency, potent in preventing infection and replication of different variants of SARS-CoV-2 variants in Vero E6 cells.

**Fig 3:**
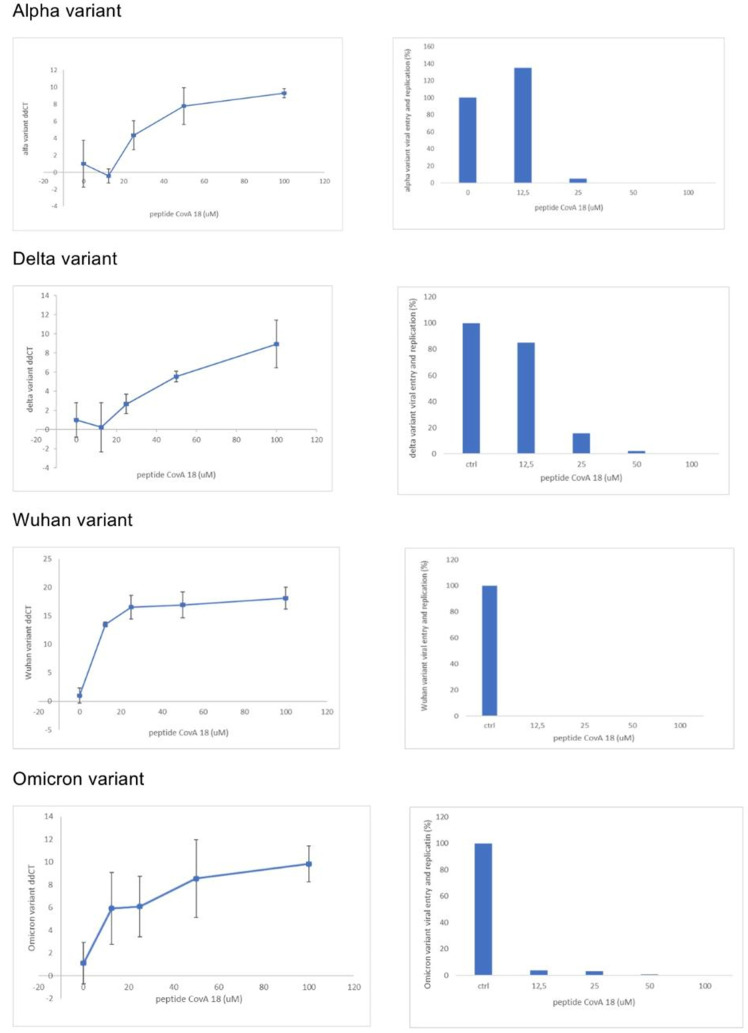
RT-qPCR determination of viral RNA for different SARS-CoV-2 variants. Viral RNA was determined with RT-qPCR targeting the E gene of SARS-CoV-2, using GAPDH as a control. Results are presented as fold change compared to non-treated cells after normalisation to GAPDH expression analysed. The Y axis on the right side represent intracellular RNA quantities in percentages of control. Peptide CovA inhibits viral entry and replication of Alpha, Delta, Wuhan and Omicron SARS-CoV-2 variants, but with different efficiency. The inhibitory activity of the peptide CovA was tested eight times at the indicated concentrations.

Furthermore, peptide CovA was tested for its inhibitory activity on viral entry and replication for Alpha, Delta, Wuhan and Omicron variants of SARS-CoV-2 virus. The peptide was added to the monolayer of washed Vero E6 cells and then infected with SARS-CoV-2. Cells were left overnight in a tested compound containing medium. Viral RNA was determined with RT-qPCR targeting the E gene of SARS-CoV-2, using GAPDH as a control. Results are presented as fold change compared to non-treated cells after normalisation to GAPDH expression analysed as described in reference [21]. Experiments have been repeated eight times and are presented in Fig 3 with relevant standard deviations. As it can be observed from Fig 3 that peptide CovA is extremely potent at the very small concentration for Wuhan and Omicron variants, while for Alpha and Delta variants the required concentration is slightly higher.

These results confirm that peptide CovA dose-dependently, with different efficiency, significantly inhibited viral entry and replication of all tested SARS-CoV-2 variants on Vero E6 cells. All raw data with statistics regarding these are presented within supporting information file S2 File.

## Discussion

Continuation of the COVID-19 disease is mostly due to occurrence of SARS-CoV-2 mutations, resulting in variations of circulating viral variants, which may impact the virus’s properties such as increased transmissibility, reduced natural or vaccine immunity [1–3]. Thus, there is a need for comprehensive approach, which should be less dependent on viral variants. For that purpose, we have used our unique, biophysical model, so called Resonant Recognition Model (RRM), to identify the common characteristics of all variants of SARS-CoV-2 virus. As identified RRM characteristics are common for all variants of SARS-CoV-2 virus, we explore here the possibility of an approach against SARS-CoV-2 infection less dependent on viral variants.

Here, it is presented that synthetic peptide CovA, designed using the RRM model, can dose-dependently with different efficiency significantly inhibit viral entry and replication of all tested SARS-CoV-2 variants on Vero E6 cells. It is important to notice that CovA peptide is designed from common RRM characteristics of all different variants of SARS-CoV-2 virus and thus expectation is that it is active against different viral variants. These target RRM characteristics are quantum physics global characteristics of the whole protein, in case here characterising SARS-CoV-2 spike protein. Thus, RRM model is quite different approach compared to existing approaches, where the targets for bioactive peptide design are certain chemical structures or chemical bonds within protein, e.g., SARS-CoV-2 spike protein. For example, the one of such approaches targets presence of disulphide bonds in the receptor binding domain of the spike protein [22]. In general, previous approaches are targeting certain chemical structures or chemical bonds, which are not very specific and may occur in many different proteins. Thus, peptides designed based on such approaches are prone to interfere with other unrelated biological processes and/or interactions. In contrast, our RRM approach is based on the whole molecule’s quantum physics global parameters within proteins, which has been shown to be specific only to target analysed biological function or interaction [7–9]. So, our RRM designed CovA peptide targets only the specific protein-receptor interaction rather than some structure or chemical bond, which can appear in many other proteins or receptors. In general, RRM designed peptides are protein-receptor interaction specific and thus, are not supposed to interfere with any other biological activity in the living body. In case here, RRM designed CovA peptide specifically interfere with interaction between SARS-CoV-2 spike protein and ACE2 receptor and is not supposed to interfere with any other biological function or interaction.

It can be observed from the experiments above that peptide CovA dose-dependently, with different efficiency, significantly inhibited viral entry and replication of all tested SARS-CoV-2 variants on Vero E6 cells. These results could be used as a starting point towards variant independent cure against SARS-CoV-2 virus. It is important to note that RRM model is capable, using quantum physics and signal processing, to analyse specific biological function and interaction of proteins and based on this analysis the RRM model is capable to design *de novo* related bioactive peptides with specific biological function.

## Materials and methods

### CovA peptide

The sequence of RRM designed CovA peptide is as follows:

HRWKQWWWAQCPYDKLDM

This peptide was synthesised by: Pepscan, Netherland, with purity 99.1% (ULCB/UV215).

### SARS-CoV-2 virus isolation

The same SARS-CoV-2 variants, as described and used in our previous study [22], were used. Brief description: the patient-derived SARS-CoV-2 strain Slovenia/SI-4265/20, D614G was provided by the European Virus Archive. SARS-CoV-2 variants Alpha/B.1.1.7 (GISAID: EPI_ISL_1240614), Delta/B.1.617.2 (AY.98.1) (GISAID: EPI_ISL_3061362), and Omicron/BA.5.2.1 (EPI_ISL_15111405) were isolated at the Institute of Microbiology and Parasitology, Veterinary Faculty, University of Ljubljana from samples collected by National Laboratory for Health, Environment and Food from SARS-CoV-2 positive individuals. Virus isolation and preparation of virus stocks for further experiments were performed on Vero E6 cells in a biosafety level 3 laboratory, as described in reference [23].

### *In vitro* cell infection

For the *in vitro* cell infection, one day before infection, Vero E6 cells were seeded into a 96-well plate at a density of 3.5x10^4^ cells per well in an Eagle’s Minimum Essential Medium (EMEM, ATCC 30−2003) containing 10% FBS (Gibco) and 1% antibiotic and antimycotic (Antibiotic-Antimycotic (100X), Gibco) at 37°C in 5%CO2 incubator, as described previously [11,22]. The inhibitory activity of the peptide CovA was tested at the indicated concentrations. The peptide diluted in EMEM was added to the monolayer of washed Vero E6 cells and pre-incubated for 1h. After pre-incubation, 100TCID_50_ of SARS-CoV-2 in EMEM were added and incubated for 1h. The virus-peptide mixture was removed, and cells were washed 2 times with EMEM. Next, 100µl of EMEM with peptide at the indicated concentrations were added to the cells and incubated for 18h. All incubations were performed at 37°C in 5%CO2 incubator.

### Viral RNA detection and quantification

The viral RNA detection and quantification was performed as already described [11,22]. Briefly: after the incubation period, the cells were washed twice with EMEM. A synthetic trypsin TrypLE™ Express Enzyme (1X) in phenol red (Gibco) was used to detach the cells, which were used for RNA isolation using the MagMAX™ CORE Nucleic Acid Purification Kit on the KingFisher Flex System (Thermo Fisher Scientific). Viral RNA was measured with the real-time RT-qPCR assay targeting the E gene of SARS-CoV-2 using primers and probes as described in reference [24]. To detect GAPDH as endogenous control, we used TaqMan Gene Expression Assays from Applied Biosystems (Thermo Fisher Scientific, assay ID Rh02621745-g). For all RT-qPCR assays, AgPath-IDTM One-Step RT-qPCR Reagents (Thermo Fisher Scientific) were used. RT-qPCR was performed by QuantStudio 5 (Thermo Fisher Scientific) using 2µl of the extracted total RNA. After the exclusion of possible outliers (determined on ∆Ct values, with the interquartile range (IQR) method that measure a spread of dispersion and represents the range within the middle 50% of data values. The results from RT-qPCR were analysed based on 2^-∆∆Ct^ method [24] with a predisposition that RNA target and GAPDH reference had similar enough qPCR amplification conditions to allow pairing.

## Supporting information

S1 File Data in relation to RRM parameters for different SARS-CoV-2 variants. (DOCX)

S2 File Experimental raw data and statistics. (XLSX)
