# Supplementary material for: Different SARS-CoV-2 variants inhibited by RRM designed peptide
Source: PLoS One. 2025 Jul 22;20(7):e0327582. doi: 10.1371/journal.pone.0327582 (PMC12282855; doi:10.1371/journal.pone.0327582)

**Data in relation to Fig 1**

The RRM model is based on findings that certain periodicities/frequencies within the distribution of energies of free electrons along the protein are strongly correlated with the protein biological function/interaction. The RRM enables these characteristic frequencies for each biological function/interaction to be identified.

When spike proteins from different coronaviruses were analysed using the RRM, the most prominent common RRM frequency has been found at f1=0.2827, as presented in Figure below:


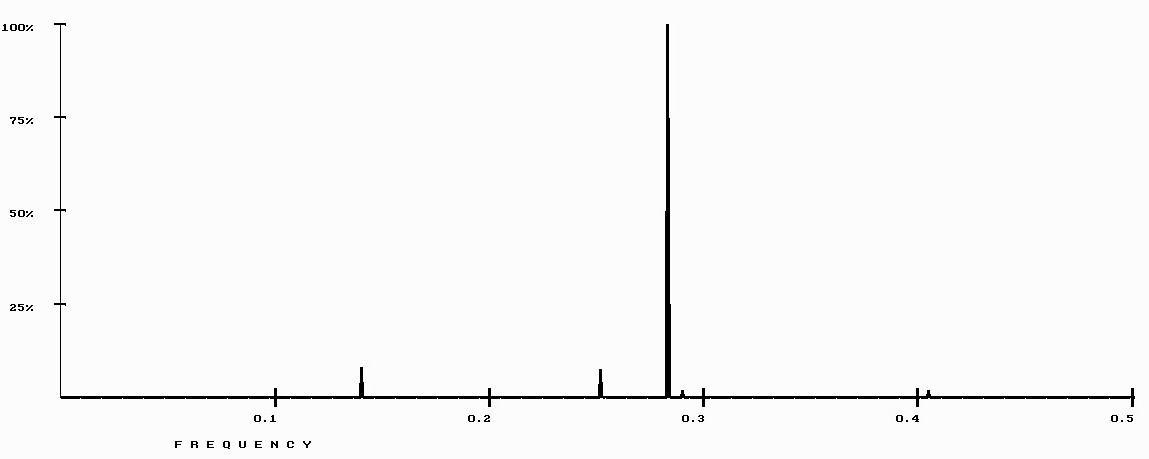


When compared, ACE2 receptors and S1 fragment of spike proteins from coronaviruses that are interacting with ACE2 receptor, the prominent common characteristic frequency appears to be at RRM frequency of f2=0.3145, as presented in Figure below:


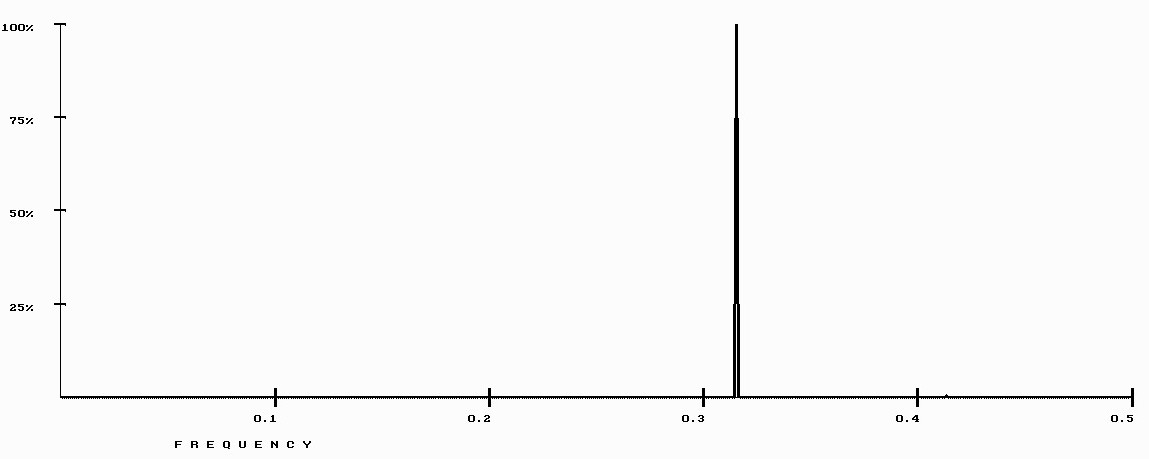


When the amplitudes (as S/N parameter) were calculated for each individual variant at two previously identified RRM characteristic frequencies f1 and f2 the results presented in Table below have been obtained:

|  | Brazilian | original | SA | Indian | UK | Delta | Omicron |
| --- | --- | --- | --- | --- | --- | --- | --- |
| f1 | 0.81 | 0.9 | 0.94 | 1.13 | 1.36 | 0.64 | 0.49 |
| f2 | 1.42 | 1.71 | 1.8 | 1.84 | 2.65 | 4.08 | 3.23 |

These results are presented graphically in Figure below, as well as in Fig 1 in the manuscript:


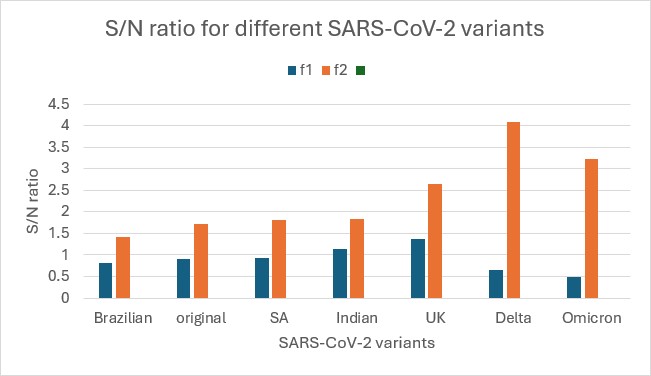


When these results are compared with the activity of variant’s SARS-CoV-2 as presented in the table below, it can be observed that more virulent variants have higher S/N ratio at RRM characteristic frequencies f2 (orange bars) and that strength of virus variant is correlated with S/N ratio at RRM characteristic frequencies f1 (blue bars).


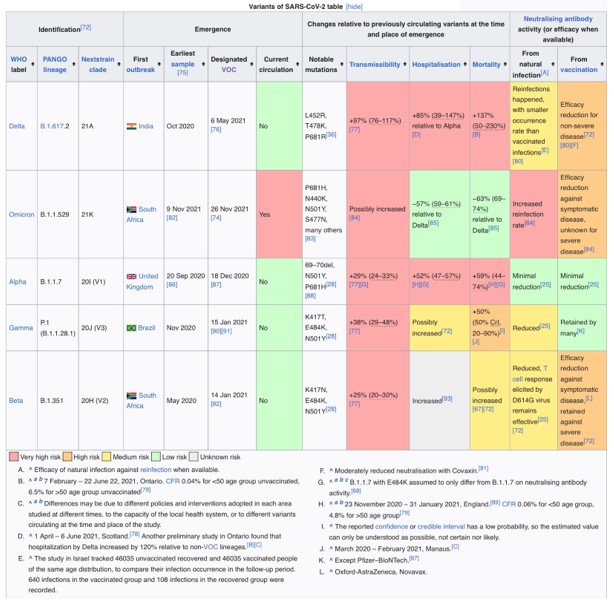

Supplement: S1 File — (DOCX) [file pone.0327582.s001.docx]
